# High glucose and high lipid induced mitochondrial dysfunction in JEG-3 cells through oxidative stress

**DOI:** 10.1515/biol-2022-0561

**Published:** 2023-02-07

**Authors:** Yang Duan, Fuqiang Sun, Yueqin Li, Suyan Yang

**Affiliations:** Department of Neonatology, Second Hospital of Tianjin Medical University, No. 23 Pingjiang Road, Hexi District, Tianjin 300211, China

**Keywords:** Nrf2/ARE signaling pathway, high lipid, high glucose, apoptosis, mitochondria

## Abstract

Few studies focused on the roles of high glucose combined with high lipid in placental development or fetal growth. This study was designed to investigate the roles of high glucose combined with high lipid in mitochondrial dysfunction of JEG-3 cells. We determined the cellular proliferation and apoptosis, superoxide dismutase (SOD) activity, concentration of malondialdehyde (MDA), and lactic acid dehydrogenase in control group, high glucose group, high lipid group, and high glucose and high lipid group, together with the mitochondrial dysfunction, Nrf2, HO-1, SMAC, and cytochrome *C* (Cyt-*C*) expression. Significant decrease of SOD and significant elevation of MDA was seen in high glucose and high lipid group compared with the other three groups. There was significant decrease in mitochondrial SMAC and Cyt-*C* in high glucose group, high lipid group, and high glucose and high lipid group compared with those of control group. Nrf2 and HO-1 protein expression in high glucose combined with high lipid group showed significant decrease compared with that of high lipid group or high glucose group. We speculated that combination of high glucose and high lipid induced oxidative stress in JEG-3 cells, and Nrf2/ARE pathway may be related to this process.

## 1 Introduction

The prevalence of maternal gestational diabetes mellitus (GDM) and obesity shows a trend of increase worldwide [1,2], and each of them has been considered as independent risk factor for poor pregnancy outcomes such as metabolic diseases in the offspring [3–5]. Up to now, our understanding on the metabolic diseases in the offspring induced by maternal hyperglycemia and obesity is still limited. It has been acknowledged that placental mitochondrial dysfunction plays crucial roles in the pathogenesis of these metabolic diseases [3]. Several factors have been reported to associate with mitochondrial dysfunction, among which oxidative stress plays an important role. Imbalance of oxidation and anti-oxidation has been frequently reported in patients with GDM and obesity, leading to excessive production of reactive oxygen species (ROS) [6,7]. Mitochondria is the major site for ROS production, and excessive generation of ROS may mediate the mitochondrial injury in the presence of oxidative stress [8,9]. The oxidative stress may induce mitochondrial dysfunction, resulting in increased membrane penetration and the subsequent release of more second mitochondria-derived activator of caspases (SMAC) and cytochrome *C* (Cyt-*C*) from mitochondria to cytoplasm [10]. On this basis, there might be a definite relationship between GDM, obesity, and mitochondrial injury mediated by oxidative stress.

Nuclear factor E2-related factor 2 (Nrf2) is a crucial element for the Nrf2-ARE signaling pathway that is important for the cells to respond to the oxidative stress [11]. Aberrant Nrf2 expression has been considered to relate to the GDM and the concurrent complications. For the mechanism, it may be related to the regulation on oxidative stress and prevention of the trophoblast injury. However, little is known about the roles of Nrf2 in mitochondrial dysfunction in the presence of high glucose and high lipid. JEG-3 cells, serving as a type of chorionic trophoblast cell line, has been frequently utilized in the development and function evaluation of placenta [12,13]. In this study, we determined the effects of high glucose and high lipid on the oxidative stress and the mitochondrial injury based on JEG-3 cells.

## 2 Materials and methods

### 2.1 Cells, reagents, and instruments

Cell counting kit-8 (CCK-8) was purchased from Sigma-Aldrich. BCA protein quantitative kit was purchased from Boster (Wuhan, China). Mouse Nrf2, HO-1, SMAC, Cyt-*C*, Lamin-B, and β-actin monoclonal antibodies were purchased from Abcam. HRP-labeled goat anti-mouse IgG antibody and goat anti-rabbit IgG antibody were purchased from Santa Cruz. Commercial kit for the determination of lactate dehydrogenase (LDH) cytotoxicity was purchased from Thermo Fisher Scientific. Western blot electrophoresis and exposure system were purchased from Bio-Rad (CA, USA) and the automatic microplate reader was purchased from Yongchuang Medical Instruments (Shanghai, China). The other facilities used in this study included cell culture plate (Corning, USA), ultra-clean workbench (Yatai Kelong Instrument, Beijing, China), centrifuge (Shanghai Lu Xiangyi Centrifuge Instrument, China), cell counting plate (Germany), and carbon dioxide incubator (USA).


**Ethical approval:** The research related to cell use has been approved by the Ethics committee of the Second Hospital of Tianjin Medical University (No. KY2021K054).

### 2.2 Cell culture

JEG-3 cells purchased from the Cell Bank of the Chinese Academy of Sciences (Shanghai, China) were cultured in DMEM medium (Gibaco) containing fetal bovine serum (10%, Gibaco). The cells were cultured in 5% CO_2_ at 37°C, and harvested until a confluence of 80%.

### 2.3 Grouping

JEG-3 cells were divided into the following groups: control group, high glucose group, high lipid group, and high glucose and high lipid group. Cells in high glucose group were cultured on DMEM medium containing 30 mmol/L glucose, and cells in high lipid group were cultured on DMEM medium containing 0.3 mmol/L palmitic acid and 5.5 mmol/L glucose. Cells in high glucose and high lipid group were cultured on DMEM medium containing 30 mmol/L glucose and 0.3 mmol/L palmitic acid. Cells in control group were cultured with DMEM medium containing 5.5 mmol/L glucose.

### 2.4 Cellular viability

Cellular viability was determined using the CCK-8 purchased from Sigma-Aldrich (Cat. No.: 96992), according to the manufacturer’s instructions. Briefly, JEG-3 cells (1 × 10^4^) in each group were incubated with 10 µL CCK solution for 3 h. The cellular proliferation was determined in each group at 0, 24, 48, and 72 h. Finally, the absorbance was measured with a microplate reader at a wavelength of 450 nm.

### 2.5 LDH leakage and cell oxidative stress level detection

Cytotoxicity assay kit (Cat. No.: ab65393) purchased from Abcam was used to measure LDH activity. A microplate reader (BioTek, USA) was utilized to measure the absorbance at 490 nm to calculate the LDH leakage. The malondialdehyde (MDA) concentration and activity of superoxide dismutase (SOD) was measured at 24, 48, and 72 h using commercial kits (Cat. No. for MDA: BC0025; Cat. No. for SOD: BC0170; Solarbio, Beijing, China) according to the manufacturer’s instructions.

### 2.6 Apoptosis analysis

For the evaluation of apoptosis, cells (5 × 10^4^) in each group were subject to trypsinization, and then were resuspended using PBS. Afterward, 5 µL Annexin V-FITC, 500 µL binding buffer, and 5 µL PI were added into the mixture, followed by incubation at room temperature in dark for 8 min. Finally, the cellular apoptosis was evaluated using flow cytometry at 24 h, according to the previous description [14,15]. Data collected from the tests were analyzed using the Kaluza system (Beckman Coulter).

### 2.7 Western blot analysis

Total protein, mitochondrial and cytoplasmic proteins were extracted from cells in each group, using commercial kit (Cat. No.: EX1320, Solarbio, Beijing, China). Protein concentration was determined using BCA method. Proteins were separated on SDS-PAGE, and then were transferred to PVDF membrane. Subsequently, the membrane was blocked using 5% skimmed milk. Then the mixture was incubated with the primary antibodies (i.e., Nrf2 and HO-1), and then were incubated with HRP-conjugated goat anti-rabbit and mice secondary antibodies. The bands were visualized using BenchPro system (Invitrogen, USA), and the band intensity of each target was quantified using Image J software. The same membrane probed with β-actin served as internal standard.

### 2.8 Immunofluorescence

The JEG-3 cells were inoculated on the petri dishes with polylysine, and then were fixed with 4% paraformaldehyde. Afterward, antigen retrieval solution (Cat. No.: AR0022, Boster, Wuhan, China) was added, followed by washing with PBS for at least three times. The goat serum blocking buffer (S-1000-20, Vector Lab, Hongkong, China) was added to the mixture, followed by incubation at 37°C for 30 min. The mixture was then incubated with Nrf2 and HO-1 monoclonal antibody (1:100). Subsequently, the goat-anti-mice secondary antibodies were added. 4',6-Diamidino-2-phenylindole was used for the nuclear staining. Finally, the images were observed under a fluorescence microscope (BX51, Olympus, Japan).

### 2.9 Determination of mitochondrial respiratory chain complex I–IV activity

Intact mitochondria isolation and activity evaluation of the mitochondrial respiratory chain complex I–IV were conducted according to the previous description [16]. Briefly, the cells were pre-treated using 50 mmol/L Tris buffer (pH = 7.5), 100 mmol/L potassium chloride, 5 mmol/L MgCl_2_, and 1 mmol/L ethylenediaminetetraacetic acid. The activity of the enzymes was determined using Beckman DU640 photometer.

### 2.10 Statistical analysis

SPSS 19.0 software was utilized for the data analysis. The measurement data were presented in a form of mean ± standard deviation. Multi-group comparison was performed using Chi-square test. Lysergic acid diethylamide method was utilized for the inter-group comparison. *P* < 0.05 was considered statistically significant.

## 3 Results

### 3.1 High glucose and high lipid inhibited proliferation of JEG-3 cells

**Figure 1:**
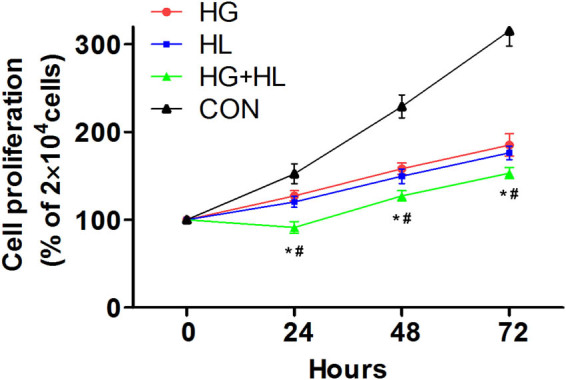
Comparison of JEG-3 cell proliferation among the four groups. *P < 0.05 versus control group, ^#^ *P* < 0.05 versus high glucose group and high lipid group.

Compared with the control group, significant decrease was observed in the proliferation of JEG-3 cells in high glucose group (*P* < 0.05), high lipid group (*P* < 0.05), especially the high glucose and high lipid group (*P* < 0.01) at 24, 48, and 72 h, respectively. Compared with the high glucose group and high lipid group, significant decrease was seen in the cellular proliferation in the high glucose and high lipid group (*P* < 0.05) at 24, 48, and 72 h, respectively. There were no significant differences in the cellular proliferation at 24, 48, and 72 h between high glucose group and high lipid group (*P* > 0.05, Figure 1).

### 3.2 High glucose and high lipid promoted LDH leakage in JEG-3 cells

**Figure 2:**
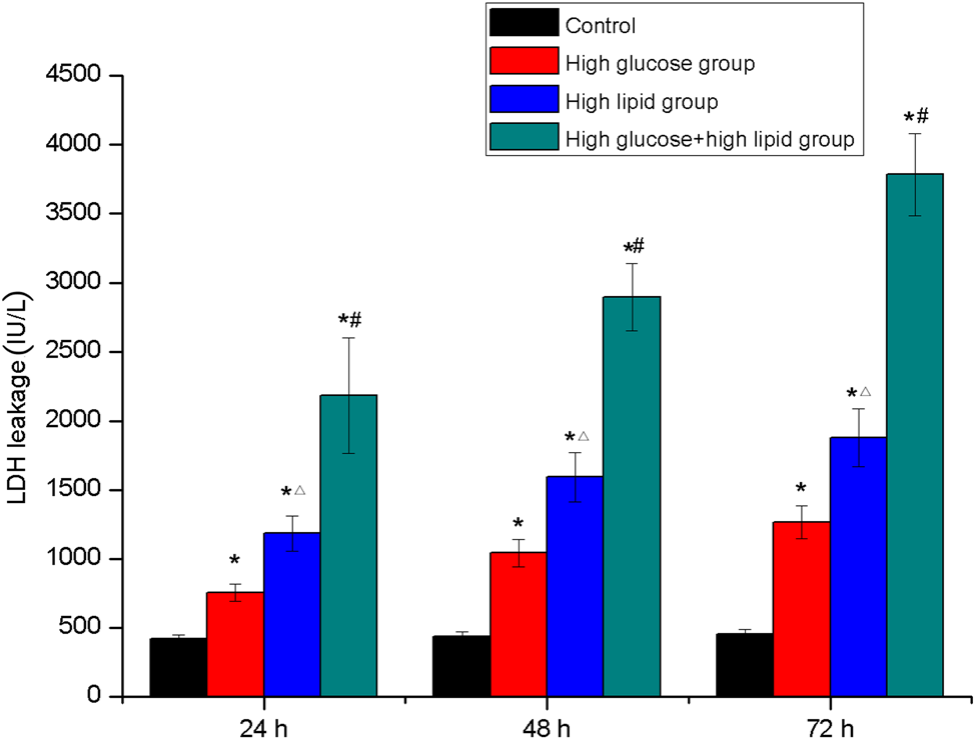
Comparison of LDH leakage among these group. *P < 0.05 versus control group, ^#^ *P* < 0.05 versus high glucose group and high lipid group, and ^△^ *P* < 0.05 versus high glucose group.

Compared with the control group, significant increase was seen in the LDH leakage in the high glucose group (*P* < 0.05), high lipid group (*P* < 0.05), especially the high glucose and high lipid group (*P* < 0.01) at 24, 48, and 72 h. Moreover, the LDH leakage in the high glucose and high lipid group was significantly higher than that of the high glucose group and high lipid group (*P* < 0.05, Figure 2). Furthermore, the leakage in high lipid group was significantly higher than that of high glucose group at 24, 48, and 72 h, respectively (*P* < 0.05).

### 3.3 High glucose and high lipid promoted apoptosis of JEG-3 cells

**Figure 3:**
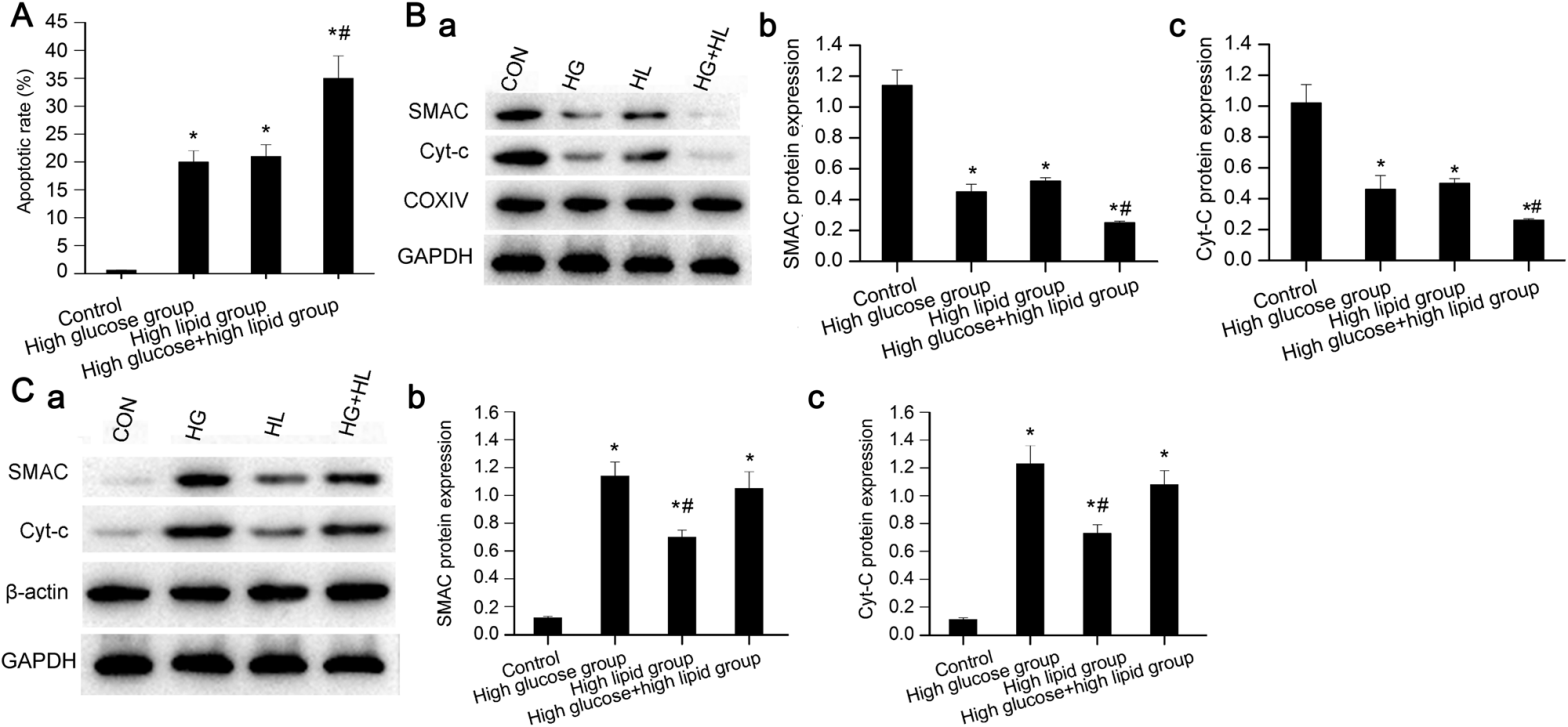
Cellular apoptosis and expression of SMAC and Cyt-*C* in mitochondria and cytoplasm. (A) Cell apoptosis in high glucose and/or high lipid groups compared with control group at 24 h. *P < 0.05 versus control group, ^#^ *P* < 0.05 versus high glucose group and high lipid group. (B) Mitochondrial SMAC and Cyt-*C* expression at 24 h based on western blot analysis. *P < 0.05 versus control group, ^#^ *P* < 0.05 versus high glucose group and high lipid group. (C) Expression of cytoplasmic SMAC and Cyt-*C* at 24 h. *P < 0.05 versus control group, ^#^ *P* < 0.05 versus high glucose group, or high glucose and high lipid group. HG, high glucose; HL, high lipid; HG + HL, the combination of high glucose and high lipid; CON, control.

**Figure A1:**
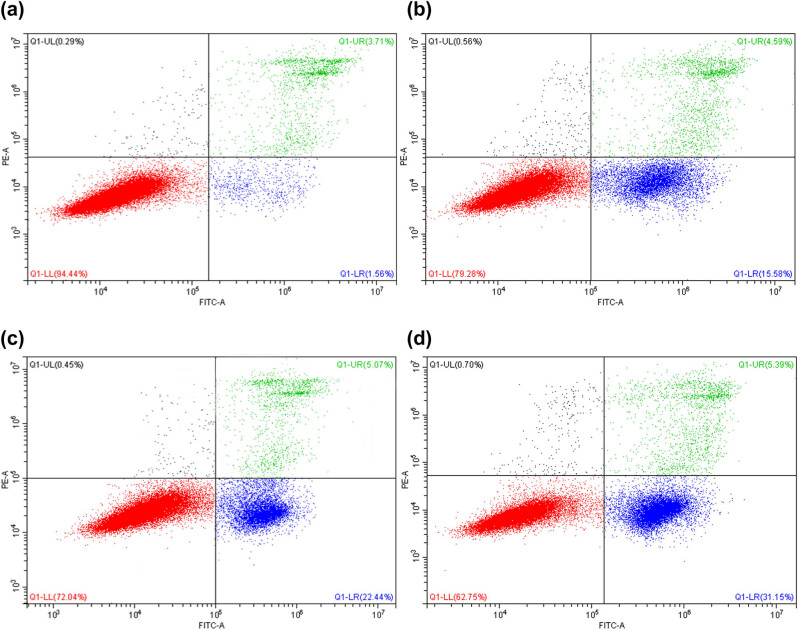
Apoptosis of JEG-3 cells (5 × 10^4^) by flow cytometry in control (a), high glucose group (b), high lipid group (c), as well as high glucose and high lipid group (d).

Flow cytometry was utilized to detect the apoptotic rate of JEG-3 cells in each group. Compared with the control group, significant increase was noticed in the proportion of apoptotic cells in the high glucose group (*P* < 0.05), high lipid group (*P* < 0.05), as well as the high glucose and high lipid group (*P* < 0.01, Figure 3A and Figure A1). Compared with the high glucose group and high lipid group, there was significant increase in the proportion of apoptotic cells in high glucose and high lipid group (*P* < 0.05). In contrast, there were no statistical differences in the apoptosis between high glucose group and high lipid group (*P* > 0.05).

In this section, we determined two important apoptotic factors including SMAC and Cyt-*C* in the JEG-3 cells. The expression of mitochondrial SMAC and Cyt-*C* protein in the high glucose and/or high lipid groups was significantly lower than that of the control group (*P* < 0.05), especially the high glucose and high lipid group (*P* < 0.01, Figure 3B). This indicated the presence of increased membrane permeability upon mitochondrial damages in the high glucose and high lipid group, which then triggered the release of apoptotic protein SMAC and Cyt-*C* into the cytoplasm from mitochondria. Subsequently, we detected the expression of cytoplasmic SMAC and Cyt-*C* protein. Compared with the control group, the cytoplasmic SMAC and Cyt-*C* showed significant upregulation in the high glucose group (*P* < 0.05), high lipid group (*P* < 0.05), as well as high glucose and high lipid group (*P* < 0.05). The cytoplasmic SMAC and Cyt-*C* in the high lipid group was significantly lower than that of high glucose group, as well as the high glucose and high lipid group (*P* < 0.05). No statistical differences were noticed in the expression of SMAC and Cyt-*C* between high glucose group and high glucose and high lipid group (*P* > 0.05, Figure 3C).

### 3.4 High glucose and high lipid triggered decrease of SOD and increase of MDA in JEG-3 cells

**Figure 4:**
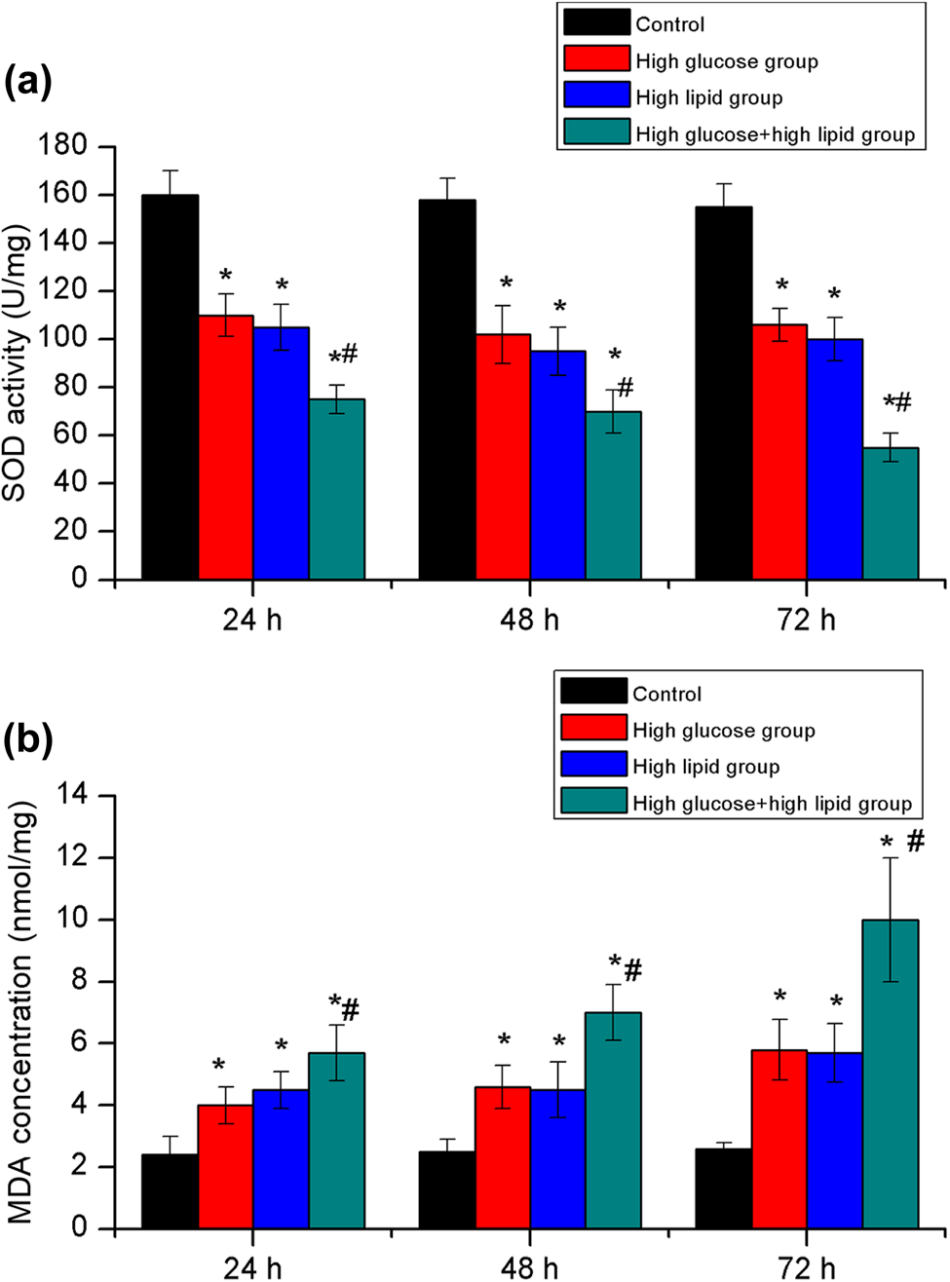
Comparison of SOD (a) activity and MDA (b) concentration among the four groups. *P < 0.05 versus control group, ^#^ *P* < 0.05 versus high glucose group and high lipid group.

In this section, SOD activity and MDA concentration in JEG-3 cells were determined in each group. At 24, 48, and 72 h, the SOD activity in the high glucose and/or high lipid groups was significantly lower than that of the control group (*P* < 0.05). The SOD activity of the high glucose and high lipid group was significantly lower than that of high glucose group and high lipid group, respectively (*P <* 0.05). In contrast, the MDA concentration of cells in the high glucose and/or high lipid groups was significantly higher than those in the control group (*P* < 0.05). The concentration of MDA in the high glucose and high lipid group was significantly higher than those in the high glucose group and high lipid group (*P* < 0.05, Figure 4).

### 3.5 High glucose and high lipid triggered decrease of mitochondrial complex I and IV activity in JEG-3 cells

**Figure 5:**
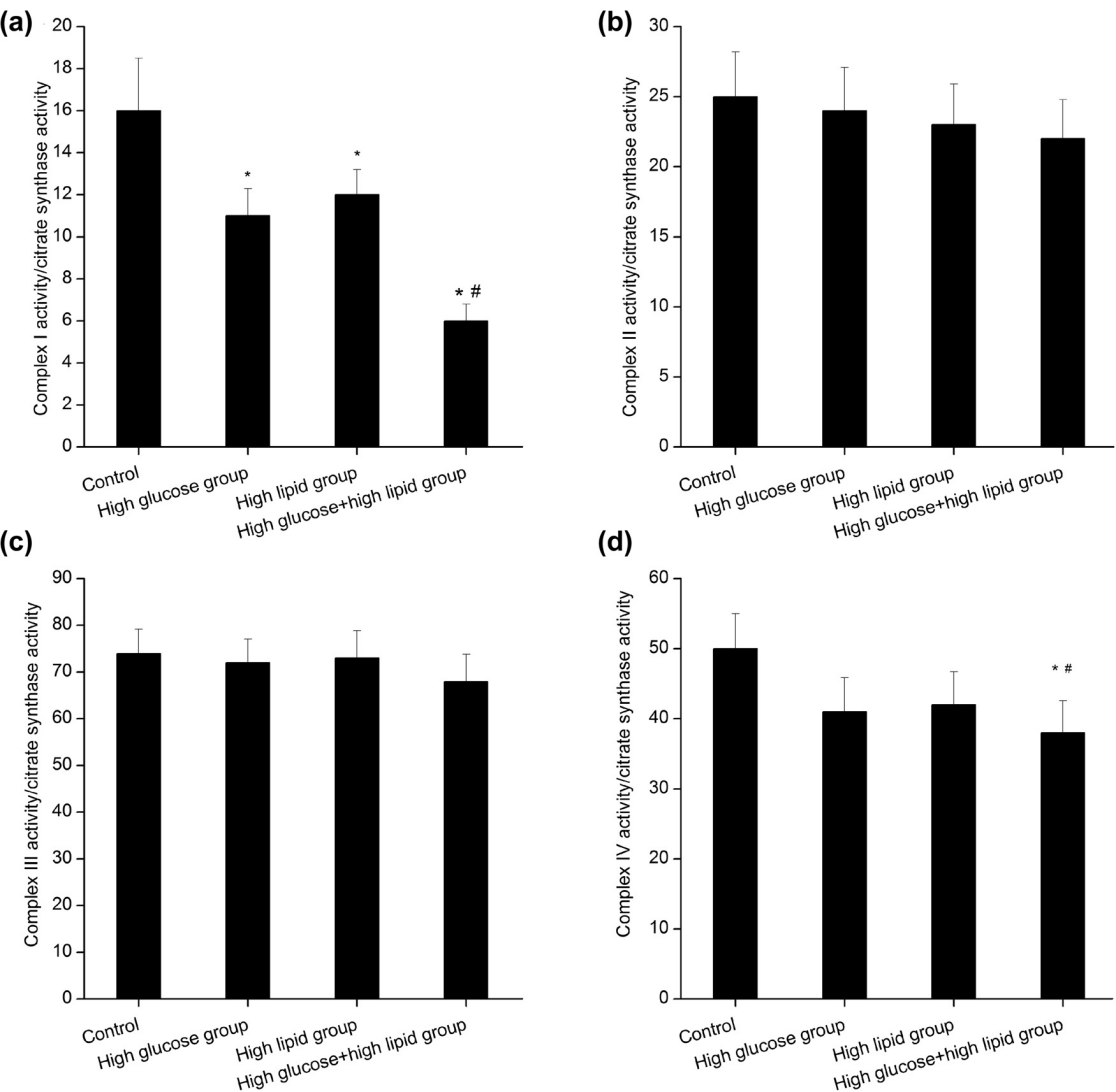
Comparison of activity of mitochondrial complex I (a), II (b), III (c), and IV (d) in different groups. *P < 0.05 versus control group, ^#^ *P* < 0.05 versus high glucose group and high lipid group.

Compared with the control group, the activity of mitochondrial complex I in JEG-3 cells showed significant decrease in high glucose group (*P* < 0.05), high lipid group (*P* < 0.05), as well as high glucose and high lipid group (*P* < 0. 01). The activity of mitochondrial complex I in high glucose and high lipid group was statistically lower than that of the high glucose group (*P* < 0.05) and high lipid group (*P* < 0.05). In contrast, there was no significant difference in the expression of mitochondrial complex I between the high glucose group and high lipid group (*P* > 0.05). No significant differences were observed in the activity of mitochondrial complexes II and III among the four groups (*P* > 0.05, Figure 5). The activity of mitochondrial complex IV in the high glucose group and high lipid group showed no statistical differences compared with that of the control group (*P* > 0.05). The activity of mitochondrial complex IV in the high glucose and high lipid group was significantly lower than that of control group (*P* < 0.05), high glucose group (*P* < 0.05), and high lipid group (*P* < 0.05). No statistical differences were noticed in the mitochondrial complex IV between the high glucose group and high lipid group (*P* > 0.05).

### 3.6 High glucose and high lipid inhibited Nrf2 and HO-1 expression in JEG-3 cells

**Figure 6:**
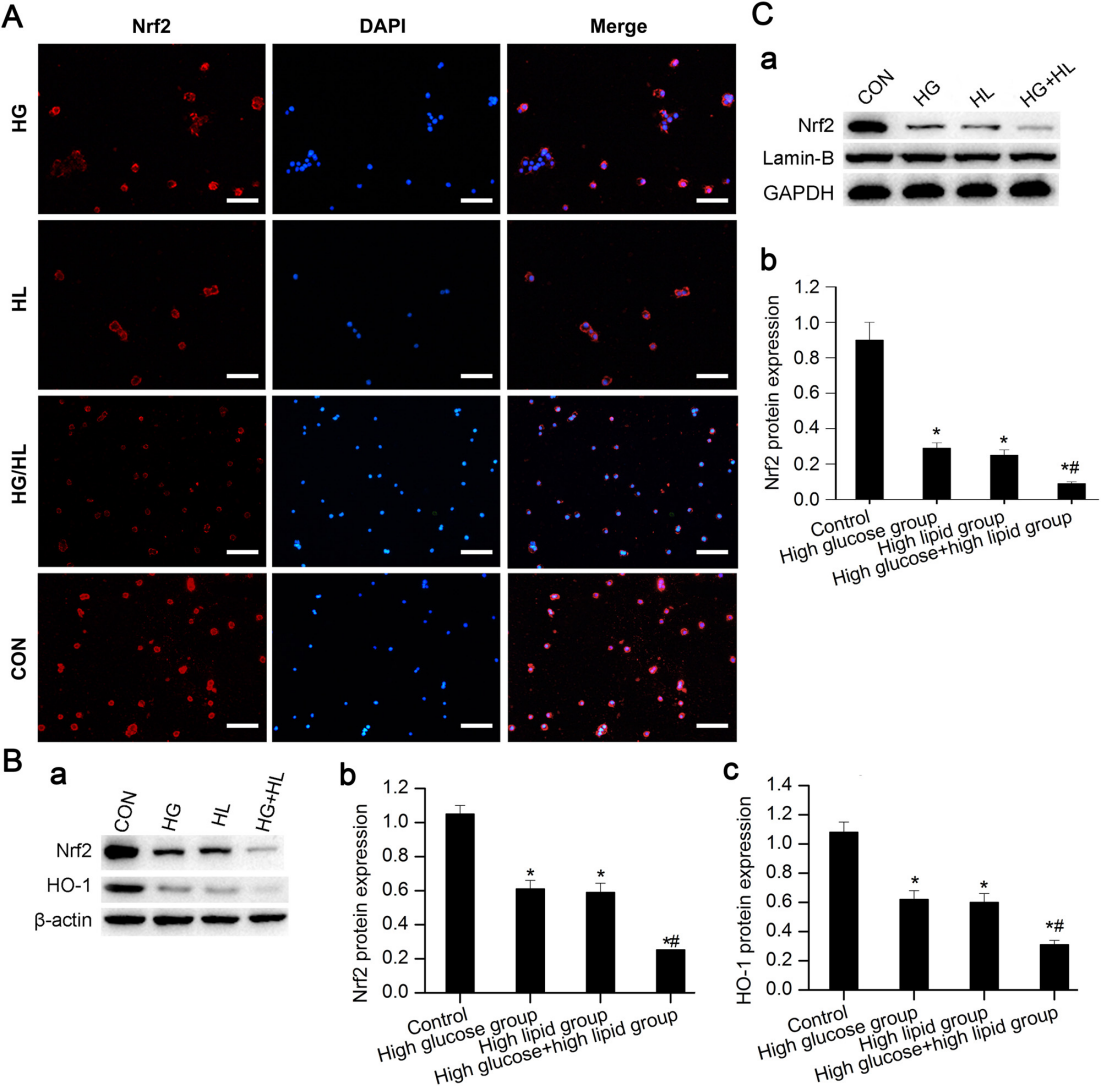
Determination of expression of Nrf2 and HO-1 in different groups. (A) Expression of Nrf2 at 24 h in each group was detected by immunofluorescence. HG, high glucose; HL, high lipid; HG + HL, the combination of high glucose and high lipid; CON, control. The bar equals to 100 µm. (B) Expression of cytoplasmic Nrf2 and HO-1 at 24 h in each group. Beta-actin served as the internal standard. *P < 0.05 versus control group, ^#^ *P* < 0.05 versus high glucose group and high lipid group. (C) Nuclear Nrf2 expression at 24 h in each group. *P < 0.05 versus control group, ^#^ *P* < 0.05 versus high glucose group and high lipid group.

Based on immunofluorescence, Nrf2 was expressed in cytoplasm and nucleus in control group, and the fluorescence intensity was higher than that of other groups. The expression of Nrf2 in cytoplasm of high glucose group and high lipid group was comparatively low, and there was no obvious nuclear Nrf2 translocation. In the high glucose and high lipid group, the expression of cytoplasmic Nrf2 was extremely lower, with no obvious Nrf2 nuclear translocation (Figure 6A). Western blot showed that compared with control group, significant downregulation was noticed in the expression of total cellular Nrf2 protein and HO-1 protein in the high glucose group, high lipid group (*P* < 0.05), and especially the high glucose and high lipid group. For the expression of Nrf2 in nuclear protein, compared with that of control group, significant downregulation was noticed in the expression of Nrf2 in high glucose group and high lipid group (*P* < 0.05), especially the high glucose and high lipid group (*P* < 0.01, Figure 6B and C).

## 4 Discussion

A large number of pregnant women (3–30%) would develop GDM and obesity according to an epidemiological survey [17], which may induce potential injuries to the placenta and embryo [18]. Placental dysfunction will directly affect the growth of embryo. Trophocytes contribute to the embryo implantation and serve as the base for nutrition supply for early-stage embryo. Therefore, we speculated that GDM and obesity could affect the proliferation, apoptosis, and cell cycle of placental trophoblasts [19]. In this study, we aimed to investigate the effects of high glucose and high lipid on the biological function of cytotrophoblast cell line JEG-3 cells.

The proliferation, differentiation, and invasion of trophocytes are necessary for placental development. In this study, we determined the proliferation of JEG-3 cells under high glucose and/or high lipid conditions. The proliferation of JEG-3 cells showed remarkable decrease in the presence of high glucose and/or high lipid, especially the combination of high glucose and high lipid. There was decline of the JEG-3 cell proliferation in high glucose group and high lipid group at 48 and 72 h. Therefore, our research fully showed that high glucose or high lipid can significantly inhibit the growth of JEG-3. Consistently, *in vitro* experiments indicated that excessive ROS may induce cellular apoptosis through the mitochondria-depending pathway if the trophoblasts were subject to excessive simulation [20–22]. In contrast, in the placental tissues of the GDM patients, the number of trophoblasts and its proliferation showed significant increase, and the apoptosis showed significant decrease. In a previous study, the proliferation of trophocytes showed significant increase in the placenta at defined developmental stages in diabetic rats [23]. In a clinical study, the placental weight of pregnant women with DM was higher than that of normal pregnant women [24], but the mechanisms were not well defined. This may be related to multiple hormones, such as the effects of placental exosome on the proliferation of trophoblasts, cell cycle, and apoptosis, as well as the placental growth factor [25].

Apoptosis is directly related to adhesion and invasion of trophoblast, and the differentiation and generation of trophoblast in the whole developmental process of human placenta [26]. The apoptotic rate of villous cytotrophoblasts and syncytiotrophoblasts in diabetic placenta was higher than the normal counterparts [27,28]. In this study, we mimicked a placental environment of high glucose and/or high glucose for JEG-3 cells, in order to investigate its effects on trophoblasts *in vitro*. The apoptotic rate in control group, high glucose group, high lipid group, and high glucose and high lipid group was 1.1, 19.5, 20.5, and 35.2%, respectively. These results indicated that high glucose could significantly promote the apoptosis of JEG-3 cells *in vitro*. In addition, the combination of high glucose and high lipid could further promote the apoptosis in the JEG-3 cells.

Hypoxia and oxidative stress could induce apoptosis of placental cells through endogenous Caspase enzyme, exogenous tumor necrosis factor, and its receptor family [29]. Endogenous damages could trigger the increase of mitochondrial membrane permeability, leading to release of Cyt-*C* into cytoplasm that could form apoptotic bodies after binding with protease activator-1 [30]. In addition, the release of SMAC protein into cytoplasm from mitochondria also contributed to the apoptosis. Upon extraction of mitochondrial proteins, the levels of apoptosis-related proteins in mitochondria (i.e., SMAC and Cyt-*C*) showed significant decrease, while the cytoplasmic expression showed significant increase. This indicated that the mitochondrial membrane permeability of JEG-3 cells increased in the presence of oxidative stress and mitochondrial injury, which then promoted the release of SMAC and Cyt-*C* into cytoplasm that finally triggered apoptosis.

In this study, the LDH leakage that was used as a biomarker for cytotoxicity and cytolysis was significantly higher in high glucose group, high lipid group, and the high glucose and high lipid group than that in the control group. Additionally, the leakage of LDH in high lipid group was also higher than that in high glucose group at 24, 48, and 72 h, respectively. The remarkable LDH leakage in the high glucose and high lipid group fully indicated that the combination of high glucose and high lipid could significantly aggravate the cellular injuries. In line with the previous studies [31,32], our data showed that the combination of high glucose and high lipid could significantly increase the oxidative stress in JEG-3 cells. In addition, the SOD activity in the high glucose and high lipid group was significantly lower than the control group, high glucose group, and high lipid group. Moreover, the MDA concentration in the high glucose and high lipid group was significantly higher than the control, high glucose group, and high lipid group. Usually, the increase of oxidative stress is often concurrent with mitochondrial injuries. Then, we detected the activity of mitochondrial complex in JEG-3 cells in each group. Consistently, the activities of mitochondrial complex I and IV in high glucose and high lipid group were significantly lower than those in the control group, high glucose group, and high lipid group.

The pathophysiological responses caused by maternal obesity and GDM are mainly associated with the oxidative stress. In this study, we explored the mechanism of oxidative stress injury of JEG-3 cells induced by high glucose combined with high lipid. Nrf-2 played important roles in the oxidative stress [33]. Under physiological conditions, Nrf2 could bind with the keap1, which then triggered the degradation of Nrf2. In the presence of oxidative stress, the keap1 was deactivated, and then would separate with Nrf2 [34]. The Nrf2 was translocalized to the nuclear, and binded with ARE, resulting in the activation of HO-1 and stability of the homeostasis [35]. Nrf2 dysregulation has been associated with the pathogenesis of the diabetes and the complications. It may protect the trophoblasts from injury through regulating the oxidative stress. Our data showed that the expression of Nrf2 and HO-1 in total protein showed significant decrease in high glucose and high lipid group. The nuclear Nrf2 expression in high glucose and high lipid group was still significantly lower than that in control group, high glucose group, as well as high lipid group. This fully indicated that the combination of high glucose and high lipid may induce mitochondrial dysfunction in JEG-3 cells, which may be related to the modulation of Nrf2/ARE signaling pathway.

There are some limitations in this study. This is an *in vitro* study mimicking the effects of high glucose and high lipid on the placental mitochondrial injuries. However, the cultured cells may not present the internal environment and regulatory effects of nerve and body fluids. In the future, we will focus on the investigation of studies using JAR and BeWo cells to illustrate the potential effects of the combination of diabetes mellitus and obesity on the placental mitochondrial dysfunction. Also, to better mimic the physiological conditions, the three-dimensional culture may be utilized in the future subsequent studies.

## 5 Conclusions

In summary, high glucose and high lipid can cause oxidative stress injuries to JEG-3 cells, and their combination would even trigger severe damages to JEG-3 cells. Nrf2/ARE signal pathway may play an important role in this biological process.
